# First Microbiological and Molecular Identification of* Rhodococcus equi* in Feces of Nondiarrheic Cats

**DOI:** 10.1155/2019/4278598

**Published:** 2019-07-09

**Authors:** Carolina Lechinski de Paula, Rodrigo Otávio Silveira Silva, Rodrigo Tavanelli Hernandes, Geraldo de Nardi Júnior, Selene Daniela Babboni, Simony Trevizan Guerra, Fernando José Paganini Listoni, Rogério Giuffrida, Shinji Takai, Yukako Sasaki, Márcio Garcia Ribeiro

**Affiliations:** ^1^Department of Veterinary Hygiene and Public Health, School of Veterinary Medicine and Animal Sciences, UNESP-São Paulo State University, Botucatu, SP, Brazil; ^2^Department of Preventive Veterinary Medicine, Federal University of Minas Gerais, UFMG, Belo Horizonte, MG, Brazil; ^3^Department of Microbiology and Immunology, Biosciences Institute, UNESP, Botucatu, SP, Brazil; ^4^Technology Faculty, FATEC, Botucatu, SP, Brazil; ^5^Paulista University, UNIP, Sao Jose dos Campos, SP, Brazil; ^6^School of Veterinary Medicine, University of Oeste Paulista, UNOESTE, Presidente Prudente, SP, Brazil; ^7^School of Veterinary Medicine, Kitasato University, Towada, Aomori, Japan

## Abstract

*Rhodococcus equi *is responsible for infections in multiple-host animals. In humans, the prevalence of rhodococcus has increased worldwide and represents an emergent risk.* R. equi* is a soil-borne opportunistic bacterium isolated from feces of a wide variety of domestic species, except cats; thus, there is no known potential risk of its transmission from humans. Here, the mono- and cooccurrence of* Rhodococcus equi* and other bacteria and selected virulence markers were investigated in feces of nondiarrheic cats from urban (n=100) and rural (n=100) areas. Seven (7/200=3.5%)* R. equi* isolates were recovered in ceftazidime, novobiocin, and cycloheximide (CAZ-NB) selective media, exclusively of cats from three distinct farms (*p*=0.01), and these cats had a history of contact with horses and their environment (*p*=0.0002). None of the* R. equi *isolates harbored hosted-adapted plasmid types associated with virulence (pVAPA, pVAPB, and pVAPN). One hundred seventy-five* E. coli* isolates were identified, and 23 atypical enteropathogenic* E. coli* (aEPEC), 1 STEC (Shiga-toxin producing* E. coli*), and 1 EAEC (enteroaggregative* E. coli*) were detected. Eighty-six* C. perfringens* type A isolates were identified, and beta-2 and enterotoxin were detected in 21 and 1 isolates, respectively. Five* C. difficile* isolates were identified, one of which was toxigenic and ribotype 106. The main cooccurring isolates in cats from urban areas were* E. coli* and* C. perfringens* A (26/100=26%),* E. coli* and* C. perfringens *type A* cpb*2^+^ (8/100=8%), and aEPEC (*eae*+/*escN*+) and* C. perfringens *type A (5/100=5%). In cats from farms, the main cooccurring isolates were* E. coli* and* C. perfringens* type A (21/100=21%),* E. coli* and* C. perfringens* type A* cpb*_2_^+^ 8/100=8%), and* E. coli* and* R. equi* (4/100=4%). We identified, for the first time,* R. equi* in nondiarrheic cats, a finding that represents a public health issue because rhodococcus has been reported in both immunosuppressed and immunocompetent humans, particularly people living with HIV/AIDS.

## 1. Introduction

The population of cats and dogs has increased worldwide, and close contact with their owners may favor the transmission of pathogens, including those with zoonotic potential, which is a public health concern [1].

Enteric pathogens are a complex group of agents represented mainly by a number of bacterial, viral, protozoal, and parasitic organisms related to infections in humans, companion animals, livestock, and wildlife [2]. These pathogens may be transmitted from cats to humans by ingestion of food, water, vegetables, and fruits contaminated with feces of their pets [1, 3].

The majority of comprehensive studies with enteric pathogens from cats involve diarrheic animals, focused mainly on one pathogen [4, 5], although diarrhea apparently is caused by a combined action of enteric pathogens [6]. Nevertheless, few studies have evaluated the complexity of the microbial population of feces from nondiarrheic cats [7, 8], as well as main virulence markers of enteric pathogens [4]. In addition, nondiarrheic cats are recognized as reservoirs of enteric pathogens [1, 4], including zoonotic agents, because these pet animals have hunting behavior and self-cleaning habits and establish broad territoriality and have close proximity with their owners [9].

Diarrheagenic* Escherichia coli *(DEC),* Clostridium, Campylobacter*, and* Salmonella* species represent the main enteric pathogens of bacterial origin that are found in normal enteric microflora [6, 10, 11] or are causal agents of diarrhea in cats [12–14].

*Rhodococcus equi* (*R. equi*) is a well-recognized bacterium that is opportunistic in nature.* R. equi *infection is characterized by diverse pyogranulomatous clinical disorders in humans, domestic animals, and wildlife [15]. It is a soil-borne organism that is widespread in the environment and is eliminated through the feces of animals [16]. In humans, the prevalence of rhodococcus has increased globally, particularly among people living with HIV/AIDS [17], and disease poses an emergent risk in the coming years [18]. This pathogen has been isolated from feces of a wide variety of animal species, including equines, bovines, small ruminants, dogs, pigs, and some wildlife. Nevertheless, to date,* R. equi* has not been isolated from the feces of cats [16], suggesting that there is a lack of a potential risk of transmission of this pathogen from cats to humans. The pathogenicity of* R. equi* is attributed to the presence of plasmid-encoded virulence-associated proteins (VAPs) [19]; however, the virulence plasmid profile of* R. equi* isolated from cats is poorly understood or neglected [20, 21].

In this scenario, the aim of the present study was to investigate the mono- and cooccurrence of* R. equi*,* E. coli *(DEC pathotypes),* Clostridium perfringens*,* Clostridium difficile*, and* Salmonella *spp. and selected virulence markers of* R. equi*,* E. coli,* and* Clostridium *species in feces of nondiarrheic cats from urban and rural areas.

## 2. Material and Methods

### 2.1. Ethics Statement

This study was performed in accordance with the Ethics Committee on Animal Use (CEUA) guidelines of the School of Veterinary Medicine and Animal Sciences, São Paulo State University, UNESP, Botucatu, SP, Brazil (protocol number 169/2014).

### 2.2. Study Design

Nondiarrheic fecal samples were collected by convenience from 100 cats from urban areas and 100 cats from rural areas and subjected to microbiological and molecular diagnostics for* E. coli*,* C. perfringens*,* C. difficile*,* R. equi*, and* Salmonella *spp. Mono- and cooccurrence of these enteric bacteria were investigated, as well as selected virulence markers of* R. equi, E. coli*, and* Clostridium* species.

### 2.3. Cats

A cross-sectional study was carried out using cats from a city and 13 farms located in the central region of the state of São Paulo, Brazil. The cats had different pedigrees, genders, and ages. The cats from rural areas had contact with bovines, equines, and small ruminants and had free access to the environment. All of the farms were similar in terms of the general management of the livestock, animal hygiene, and facilities. The exclusion criteria were as follows: (1) cats with clinical signs of diarrhea or other clinical manifestations and (2) cats undergoing antimicrobial therapy or those with history of antimicrobial use in the last 10 days.

### 2.4. Fecal Samples

Fresh fecal material was collected directly from the rectums of cats using cotton swabs and immediately introduced in Stuart's media. The samples were transported to the laboratory in plastic containers under refrigeration (4-8°C). At the laboratory, the samples were diluted in 5 mL of sterilized distilled water, and aliquots were stocked frozen (-20°C) for further microbiological and molecular analyses. Fecal samples were collected from each cat only once.

### 2.5. Microbiological Identification of E. coli, Salmonella spp., and R. equi

For* E. coli *isolation, fecal samples were plated on MacConkey agar and defibrinated sheep blood agar (5%), incubated in aerobic conditions at 37°C, and evaluated at 24, 48, and 72 hours [22]. For* Salmonella *isolation, fecal samples were inoculated into selective tubes containing tetrathionate broth and then incubated overnight in aerobic conditions at 37°C. After incubation, samples were cultured on Salmonella/Shigella (SS) agar plates and incubated aerobically at 37°C for 24-48 hours [22]. Colonies with black central areas in SS agar compatible with* Salmonella* were subjected to a commercial agglutination test using a polyvalent anti-*Salmonella *serum (Probac™, Sao Paulo, Brazil). For* R. equi *isolation, fecal samples were cultured in ceftazidime, novobiocin, and cycloheximide (CAZ-NB) selective media incubated in aerobic conditions at 37°C and evaluated at 24, 48, and 72 hours [23, 24]. All microorganisms were classified using conventional phenotypic tests [22].

### 2.6. Diarrheagenic E. coli Pathotypes Identification

The* E. coli* isolates were cultured overnight in Luria Bertani (LB) agar. Later, 3 to 5 bacterial colonies were resuspended in 200 *μ*L of sterile water and boiled for approximately ten minutes for DNA extraction. Virulence factor-encoding genes, routinely used for classification of the* E. coli* isolates in the distinct DEC pathotypes, were searched for PCR using primers and conditions as previously described [25, 26]. PCR was performed using the GoTaq Green Master Mix with 0.34 *μ*M of each of the primers and 1.0 *μ*L of bacterial DNA (Promega, Madison, WI, USA). The following positive controls were used for PCR: typical EPEC (enteropathogenic* E. coli*) E2348/69 (*eae*^+^/*escN*^+^/*bfpA*^+^/*bfpB*^+^), STEC (Shiga toxin producing* E. coli*) O157:H7 EDL933 (*eae*^+^/*stx1*^+^/*stx2*^+^), ETEC (enterotoxigenic* E. coli*) H10407 (*elt*^+^/*est*^+^), EIEC (enteroinvasive* E. coli*) EDL1284 (*ipaH*^+^), and EAEC (enteroaggregative* E. coli*) 042 (*aatA*^+^/*aagR*^+^). The C600 laboratory* E. coli* sample was used as a negative control in all PCRs. Notably, typical (tEPEC) and atypical (aEPEC) EPEC were differentiated based on the presence of the* bfpA* and/or* bfpB* genes, respectively, located in the EAF (EPEC adherence factor) plasmid, only in the formed group [27, 28].

### 2.7. Molecular Identification and Virulence of R. equi

*Rhodococcus equi* isolates were detected by using a polymerase chain reaction (PCR) [29]. Plasmid DNA from* R. equi* was obtained by the alkaline lysis method [29]. The extracted genetic material was analyzed by digestion with restriction enzymes (HindIII, BamHI, EcoRI, and EcoT22). Then, the plasmid samples were separated by electrophoresis and examined under UV light. PCR for the genes* vapA*,* vapB*, and* vap*N was performed as previously described [19, 29].

### 2.8. *Clostridium difficile* Isolation, PCR, A/B Toxin Detection, and Ribotyping

To isolate* C. difficile* spores, equal volumes of the diluted stool samples and 96% ethanol (v/v) were mixed; after incubation for 30 minutes at room temperature, 50 *μ*L aliquots were inoculated on plates containing cycloserine-cefoxitin fructose agar supplemented with 7% horse blood and 0.1% sodium taurocholate (Sigma-Aldrich Co., St. Louis, MO, USA). After anaerobic incubation at 37°C for 96 hours, all colonies with suggestive morphology were subjected to a previously described multiplex-PCR for a housekeeping gene (*tpi*), toxins A (*tcdA*) and B (*tcdB*), and a binary toxin gene (*cdtB*) [30]. All toxigenic* C. difficile* isolates were submitted to PCR ribotyping. Intergenic spacer regions were amplified using Bidet primers as previously described [31]. Amplification products were separated by electrophoresis in 3% agarose gel (Bio-Rad, California, USA) for 5 hours at 2.5 V/cm, and the gel was analyzed with BioNumerics 7.00 (Applied Maths, Belgium). PCR ribotypes were designated by international Cardiff nomenclature.

### 2.9. Isolation and Genotyping of Clostridium perfringens

To isolate* C. perfringens*, 50 *μ*L of diluted feces was inoculated in 10 mL of BHI broth. After incubation at 37°C for 24 hours, 10 *μ*L of the culture was plated onto SPS agar (SPS, Difco Laboratories, USA) and anaerobically incubated at 37°C for 24 hours [32]. After incubation, at least three typical colonies from each dilution were subjected to a previously described PCR protocol [33] for the detection of genes encoding the major* C. perfringens* toxins (alpha, beta, epsilon, and iota), beta-2 toxin (*cpb2*), and enterotoxin (*cpe*). For the detection of the NetB-, NetE-, NetF-, and NetG-encoding genes (*netB*,* netE*,* netF*, and* netG*, respectively), PCR protocols described by Keyburn et al. (2008) and Gohari et al. (2015) [34, 35] were applied.

### 2.10. Statistical Analysis

Statistical analysis was performed using R (version 3.2.3) software. The chi-square test with Yates correction (or Fisher's exact test) was used to compare the frequencies of the different enteric pathogens between cats from urban and rural areas and to compare the presence of different virulence markers among the two groups of animals. Odds ratios (ORs) and 95% confidence intervals (CIs) were calculated for positive results by area. The statistical significance level was set at 0.05.

## 3. Results

### 3.1. Mono- and Cooccurrence

The overall prevalence of bacteria isolated from feces was 87.5% (175/200)* E. coli *isolates, 43% (86/200)* C. perfringens *type A, 3.5% (7/200)* R. equi*, and 2.5% (5/200)* C. difficile. *No* Salmonella *spp. isolates were identified in the sampled cats (Table 1).

The most frequent cooccurrence of microorganisms and selected virulence factors in cats from urban areas were as follows: 26% (26/100)* E. coli* and* C. perfringens* type A, 8% (8/100)* E. coli* and* C. perfringens *type A* cpb*2^+^, and 5% (5/100) atypical EPEC and* C. perfringens *type A. Those in cats from farms were as follows: 21% (21/100)* E. coli* and* C. perfringens* type A, 8% (8/100)* E. coli* and* C. perfringens *type A* cpb*_2_^+^, 4% (4/100)* E. coli* and* R. equi*, and 3% (3/100)* E. coli* and nontoxigenic* C. difficile* tcdA^−^/tcdB^−^/cdtB (Table 2).

### 3.2. E. coli

We identified 175 (175/200=87.5%)* E. coli* isolates from cats from urban (93 isolates) and rural (82 isolates) areas (OR=2.91). The* escN* gene was detected in 13.0% (23/175) of the isolates. All 23 isolates were negative for the* bfpA* and* bfpB* genes and were thus classified as aEPEC. In addition, one STEC (harboring* stx2 *gene) and one atypical EAEC (*aatA*^+^/*aggR*^−^) were identified. The risk of positive* E. coli* isolation in urban cats was 2.91 (OR=2.91) times greater than cats from rural areas.

### 3.3. Clostridium Species

Eighty-six (86/200=43%)* C. perfringens* type A isolates were isolated (Table 1). Twenty-one (21/86=24%) of these isolates were positive for the beta-2 toxin-encoding gene (*cpb*_2_), while one isolate (1/86=1.1%) was positive for the enterotoxin-encoding gene (*cpe*). Five (5/200=2.5%)* C. difficile* isolates were identified, but only one isolate was toxigenic positive for A and B toxin-encoding genes.

### 3.4. R. equi

Seven (7/200=3.5%)* R. equi* isolates were isolated from feces in CAZ-NB selective media exclusively among cats from farms (Table 1). All these cats had contact with horses and their breeding environment, showing a statistical association between* R. equi *isolation and rural areas (*p*=0.0002). None of* R. equi* isolates harbored three hosted-adapted plasmid types associated with virulence (pVAPA, pVAPB, and pVAPN) and were considered plasmidless or avirulent isolates.

## 4. Discussion

The increase in the number of dogs and cats and their importance to the physical and mental health of their owners has been highlighted globally. The close relationship and direct contact between these animals and their owners are essential to understanding the epidemiology and virulence of different pathogens that affect pet animals and to the implementation of control strategies to avoid transmission of diseases from pets to humans [1]. In particular, over 40 million cats share households in Brazil. In this scenario, we investigated the mono- and cooccurrence of different bacteria and selected virulence markers in feces from nondiarrheic cats from Brazil. The presence of* E. coli*,* C. perfringens*,* C. difficile*, and* R. equi* harboring different virulent markers and/or toxins from feces of nondiarrheic cats from urban and/or rural areas highlights the risk of transmission of these pathogens from cats to humans due to the close contact of owners and their cats. In addition, we report the first microbiological and molecular identification of* R. equi* from the feces of cats, where the virulence plasmid profile was assessed.

The majority of comprehensive studies that have investigated the prevalence of enteric pathogens among cats have involved diarrheic animals, particularly focused on only one pathogen [4, 5]; however, diarrhea is apparently caused by the combined action of agents from the enteric microbiota of cats [3, 6]. In addition, enteric disorders are influenced by diverse factors, such as age, pedigree, history of vaccination for some pathogens, and nutritional, environmental, and management conditions [6]. Conversely, few studies have evaluated the complexity of the microbial population from feces of nondiarrheic cats [7, 8]. In the current study,* E. coli* (175/200=87.5%) and* C. perfringens* type A (86/200=43%) were the main enterobacteria isolated from nondiarrheic cats, whereas* R. equi *(7/200=3.5%) and* C. difficile* (5/200=2.5%) were isolated in lower frequencies, and no* Salmonella *species were identified. This result indicates that* E. coli* and* C. perfringens* type A are major agents of bacterial origin found in the enteric microbiota of nondiarrheic cats [4, 7, 8].

The most frequent cooccurring microorganisms (and selected virulence markers) in the nondiarrheic cats sampled were aEPEC and* C. perfringens* (type A and* cpb*2^+^). This result is not surprising because enterobacteria and clostridia are major groups of bacteria inhabitants of the enteric tract of domestic animals [22]. In addition, this finding reinforces the complexity of microorganisms present in feces of nondiarrheic cats, including zoonotic pathogens. This finding poses a public health concern due to the potential risk of transmission of fecal pathogens from cats to humans by ingestion of contaminated food, water, vegetables, and fruits [1, 3], apparently favored by some behaviors of cats, for example, hunting and self-cleaning habits, establishment of broad territoriality, and close proximity with their owners [9].

The pathogenicity of enteric and extraenteric* E. coli* involved in human, livestock, pets, and wildlife infections is characterized by a diversity of virulence factors, particularly exotoxin production, adherence to epithelial cells, iron uptake, and serum resistance [36]. In the current study, 175* E. coli* strains were isolated from nondiarrheic cats, with an OR=2.91 in urban (93 isolates) and rural (82 isolates) areas. These strains were subjected to diagnostics for selected virulence markers of typical EPEC (*eae*,* bfp*A,* bfp*B), atypical EPEC (*esc*N), EHEC (*stx*1,* stx*2), ETEC (*elt*,* est*), and EAEC (*aat*A,* aag*R) pathotypes related to human infections. Of these, the genes* eae*,* esc*N,* stx*2, and* aat*A were detected in the isolates, a finding that indicates the potential transmission of DEC pathotypes to humans because they may be harbored by domestic cats.

tEPEC and aEPEC are epidemiologically related to diarrhea in humans [27, 28, 36]. These strains contain a pathogenicity island (PAI) named locus enterocyte effacement (LEE). In our study, the* eae* and* esc*N genes used for identification of the LEE region in the* E. coli* isolates were concomitantly identified in 13.0% of our nondiarrheic cats, indicating that these animals act as an important reservoir of aEPEC. In France,* E. coli* isolates harboring the* eae* gene were reported in 5% of feces from cats with and without diarrhea [37]. Likewise, in another study performed in Brazil, this gene was observed in isolates obtained from 2.5% of the nondiarrheic cats studied [5]. Nevertheless, the presence of aEPEC isolates in our nondiarrheic cats indicates a public health concern due to the association of this pathotype of DEC with enteric diseases in humans [36].

STEC serotype O157:H7 has become important as a cause of foodborne and waterborne disease in humans that is eliminated in feces of animals, although the impact of cats in the transmission of STEC to humans remains unclear [5]. This potentially life-threatening illness produces high morbidity and mortality rates, particularly in children, and causes acute to chronic thrombocytopenia, hemolytic anemia, and hemorrhagic colitis [38].* stx*1 and* stx*2 (formerly verocytotoxin or Shiga toxin) are the main virulence factors of STEC. The gene* stx*2 was detected at low frequency among cats, in 0.6% of urban areas animals. Likewise, virulent stx-producing* E. coli *was reported in a cat from Argentina [39]. The detection of* E. coli* harboring* stx* genes without the LEE region in feces from our nondiarrheic cats is circumstantial evidence that these pet animals may represent potential carriers of STEC, which may be transmitted to their owners.

The genes* aat*A and* aag*R constitute virulence markers of the EAEC pathotype, which is related to persistent diarrhea in children, travelers, and human patients living with HIV/AIDS [40]. Nevertheless, the impact of the EAEC pathotype in companion animals remains unclear [41]. Indeed, only one* E. coli *strain was positive for the* aat*A gene, although this isolate lacks the* aggR* gene, which is responsible for encoding a member of the AraC/XylS family of bacterial transcriptional activators. None of the* E. coli *strains isolated from our 200 nondiarrheic cats were positive for the* elt* and* est *genes, which are considered virulence markers of the ETEC pathotype [36].

Clostridia are well-known Gram-positive spore-forming bacilli characterized by anaerobic requirements and the production of potent hemolytic, neurotoxic, and histotoxic entero- and exotoxins that affect humans and animals [22]. In companion animals, this bacterium causes hemorrhagic and necrotic enteritis.* Clostridium *species are widely distributed in soil and water, although many pathogenic species are normal inhabitants of the enteric microflora of humans and animals. In particular,* C. perfringens *stands out due to its capacity for toxin production. This bacterium is classified into five types (A to E) according to the production of four major toxins: alpha, beta, epsilon, and iota [14]. In addition to these major toxins, it can produce other virulence factors that are associated with the pathogenesis of some diseases in humans and animals, such as enterotoxin, which is responsible for foodborne disease in humans, and NetF, which is responsible for bloody diarrhea in dogs [35].

Among our 200 nondiarrheic cats, eighty-six (43%)* C. perfringens* type A isolates were identified, without a statistical association with urban or rural areas. In a cross-sectional study of 100 cats admitted to a municipal shelter in the USA,* C. perfringens *was identified in 42% and 50% of animals with and without diarrhea, respectively [7]. In the UK, a prevalence of 56.6% of* C. perfringens *was reported among 11151 samples from diarrheic cats submitted to a reference veterinary laboratory [8].

In the current study, the beta-2 encoding gene (cpb2) was the most prevalent and was detected in 24% of isolates from 10 and 11 cats from urban and rural areas, respectively. In previous studies, the prevalence of* C. perfringens cpb*_2 _was between 10% and 30% in dogs and cats [14]. Although some authors suggest that beta-2 toxin is relevant for the pathogenesis of* C. perfringens*-induced infections in some species, its real role is still poorly understood [42]. One of our isolates (1.1%) from an urban cat was also positive for the enterotoxin-encoding gene (*cpe*). Although* C. perfringens cpe*+ is responsible for foodborne disease and nosocomial diarrhea in humans, the role of domestic animals as reservoirs of these strains is unclear (Uzal et al., 2014) [42].* C. perfringens cpe*+ is also associated with bloody diarrhea in dogs when also positive for the recently described NetF-encoding gene (*net*F) [43]. All* C. perfringens* isolates were negative for NetF, and, thus far, these strains have been described only in dogs and foals with gastrointestinal disorders [35, 43].

*C. difficile *is a highly pathogenic species in humans related to gastrointestinal manifestations secondary to nosocomial infections and prolonged use of antimicrobials. It is also linked to diarrhea in dogs, but its role in cats remains unclear [43]. Toxigenic* C. difficile* strains produce A and/or B toxins encoded by the genes* tcd*A and* tcd*B, respectively [44], as well as binary toxin (CDT), which is related to some ribotypes, including pet animals [45]. Five (2.5%) isolates of* C. difficile* were identified in the sampled cats, but only one was toxigenic (A+B+C+D). Notably, this strain was ribotype 106, a common ribotype related to* C. difficile* in humans worldwide [46–48]. The role of cats as reservoirs for toxigenic* C. difficile* is poorly understood, as opposed previous studies have suggested that dogs may be a potential source of this bacterium for humans [49].

Salmonellosis is one of the most common zoonosis globally.* Salmonella enterica *possesses different pathogenic serotypes in humans and domestic animals. This opportunistic bacterium may be eliminated in feces of domestic animals with and without enteric signs [50]. No* Salmonella *species were isolated from our 200 nondiarrheic cats. Other studies have reported a low prevalence [7, 8] or absence [6] of isolation of* Salmonella *species in feces of diarrheic and nondiarrheic cats. Nevertheless, despite the lack of isolation of* Salmonella *in the sampled cats, due to the high pathogenicity of this agent in animals and humans as well as the risk of fecal elimination among apparent healthy or nondiarrheic animals, it is recommended that this pathogen be considered in the diagnosis of enteric agents from pet animals.

*R. equi* is a well-recognized Gram-positive bacterium that is opportunistic in nature, is characterized by various pyogranulomatous clinical disorders, and is able to infect humans, livestock, companion animals, and wildlife [15]. In humans, clinical rhodococcus has increased worldwide, and the majority of cases occur among immunosuppressed patients, especially people living with HIV/AIDS [20]. Nevertheless, clinical disease in immunocompetent humans has also been reported [17, 29]. It is a soil-borne organism that is widespread in the environment and eliminated through the feces of different animal species. However, to date,* R. equi* has not been isolated from the feces of cats [16], and the risk of transmission of this pathogen from cats to humans remains unclear [16]. Curiously, fecal samples from seven of our 200 cats showed isolation of* R. equi *in selective culture media, which was confirmed by molecular methods. The successful isolation of* R. equi* may be attributed to the efficacy of ceftazidime, novobiocin, and cycloheximide, which are included in CAZ-NB media to inhibit the growth of other microflora found in the feces of cats because these selective media have been indicated to isolate pathogens in contaminated material from foals, pigs, and farm environments [23, 24]. In addition, isolation of* R. equi *exclusively from cats from rural areas (p=0.0002) may be due to contact with livestock species because these pathogens are eliminated in feces (particularly equines and bovines) and are widely distributed in farm environments [16, 17]. To our knowledge, this report describes, for the first time, the identification of* R. equi* in the feces of nondiarrheic cats, indicating that this pet animal, particularly cats from rural areas, may be a reservoir of this pathogen for human and other animal species.

The pathogenicity of* R. equi* is characterized by plasmid-governed infectivity attributed to the presence of VAPs. Three host-adapted virulence plasmid types are recognized: pVAPA, pVAPB, and pVAPN [19]. The pVAPA type is harbored by isolates that cause typical life-threatening pyogranulomatous bronchopneumonia and ulcerative colitis in foals [17], as opposed to pVAPB type strains that are mainly recovered from the lymph nodes of pigs and wild boars [24] and human* R. equi*-induced infections, especially among immunocompromised patients [20]. The novel pVAPN, which was recently described, has been recovered from the lymph nodes of slaughtered cattle and humans, including people living with HIV/AIDS [51]. In addition,* R. equi* lacks the* vapA*,* vapB*, and* vapN* genes and is mainly isolated in the feces of livestock and farm environments. These strains are called “plasmidless” or “avirulent” [17, 19]. Conversely, a restrict number of studies have focused on investigating the virulence plasmid pattern of* R. equi* isolated from cats [20, 21]. In this context, characterization of the virulence of* R. equi* strains of nine cats and nine dogs presenting diverse clinical manifestations from Canada, USA, South Africa, Brazil, and New Zealand revealed that five feline isolates and one canine strain harbored pVAPA (formerly VapA), and the 12 remaining isolates were considered avirulent (plasmidless) [20]. A report of cutaneous lesions caused by* R. equi* in a 2-year-old male domestic cat from Brazil revealed that the isolate carried the pVAPA type (87-kb type I variant) [21]. In the current study, none of the* R. equi *isolates harbored hosted-adapted plasmid types associated with virulence (pVAPA, pVAPB, pVAPN). In addition to these seven cat isolates currently classified as plasmidless or avirulent, they hypothetically might harbor unknown virulence plasmid types [51]. Surveillance studies concerning the hosted-adapted plasmid pattern of* R. equi* strains isolated from domestic animals from different geographic areas and countries are important for investigating the virulence and transmission risks of this pathogen from animals to humans, particularly because clinical rhodococcus has been reported in both immunocompromised and immunocompetent human patients, including disease caused by plasmidless* R. equi *strains [20, 52].

## 5. Conclusions

The presence of* R. equi*,* C. perfringens*,* C. difficile*, and diarrheagenic* E. coli* harboring virulent markers in feces of nondiarrheic cats from urban and/or rural areas highlights the risk of transmission of these zoonotic pathogens from cats to humans due to close contact of owners and their cats. Moreover, we report the first microbiological and molecular identification of* R. equi* from the feces of nondiarrheic cats, providing evidence that this pet animal, especially cats from rural areas, constitutes a reservoir of this pathogen for human and other animal species.

## Figures and Tables

**Table 1:** Frequency of bacteria isolates of 200 nondiarrheic domestic cats from urban (n=100) and farm (n=100) areas. Brazil, 2014-2015.

Microorganisms	*Escherichia coli*		*Rhodococcus equi*		*C. perfringens* A		*Clostridium difficile*	
	Negative	Positive	Negative	Positive	Negative	Positive	Negative	Positive
Areas	n (%)	n (%)	n (%)	n (%)	n (%)	n (%)	n (%)	n (%)
Urban	7 (7)	93 (93)	100 (100)	0 (--)	54 (54)	46 (46)	98 (98)	2 (2)
	CL (0.880-0.980)		CL*∗*		CL (0.362-0.558)		CL*∗*	
Farm	18 (18)	82 (82)	93 (93)	7 (7)	60 (60)	40 (40)	97 (97)	3 (3)
	CL (0.795-0.895)		CL (0.02-0.120)		CL (0.304-0.446)		CL*∗*	
Total	25 (12)	175 (88)	193 (96)	7 (4)	114 (57)	86 (43)	195 (97.5)	5 (2.5)
*p* value	0.03^a^		0.01^b^		0.5^a^		1.0^b^	

n: number of isolates; %: percentage; CL: Confidence Limits; CL*∗*: not calculated due to absence of microorganisms or low number of positives; *C. perfringens* A: *Clostridium perfringens* A

*p* values less than 0.05 indicate significant differences between the urban and farm areas for the same pathogen (Chi-square^a^ or Fisher Exact^b^ tests).

**Table 2:** Mono- and cooccurrence of bacteria and selected virulence markers identified in 200 nondiarrheic domestic cats from urban and farm areas. Brazil, 2014-2015.

	Areas	Urban		Farm		
Microorganisms		n	(%)	N	(%)	p value
*E. coli∗*		41	(41)	34	(34)	0.380
*C.perfringens* A*∗*		4	(4)	5	(5)	1.000
*R. equi∗*		0	(0)	1	1.0	1.000
*C. perfringens* A *cpb*_2_^+^		0	(0)	2	(2)	0.400
*C. difficile tcd*A^+^,* tcd*B^+^, *cdt*B^−^		1	(1)	0	(0)	1.000
aEPEC *(eae*^+^, *esc*N^+^)		9	(9)	4	(4)	0.200
*E. coli* ^*∗*^+* C. perfringens *A*∗*		26	(26)	21	(21)	0.500
*E. coli∗*+* C. perfringens* A *cpb*_2_^+^		8	(8)	8	(8)	1.000
*E. coli∗*+* C. perfringens *A* cpe*^*+*^		0	(0)	1	(1)	1.000
*E. coli∗*+ *R. equi∗*		0	(0)	4	(4)	0.120
*E. coli∗*+* C. difficile*^*∗*^		1	(1)	3	(3)	0.600
aEPEC (*eae*^+^, *esc*N^+^) + *C. perfringens* A*∗*		5	(5)	1	(1)	0.200
aEPEC (*eae*^+^, *esc*N^+^)+* C. perfringens *A* cpb*_2_^+^		1	(1)	1	(1)	1.000
aEPEC (*eae*^+^, *esc*N^+^) + *R. equi∗*		0	(0)	2	(2)	0.400
STEC(*stx*2^*+*^)+ *C. perfringens* A*∗*		1	(1)	0	(0)	1.000
aEAEC (*aatA*^+^/*aggR*^−^)+* C. perfringens *A* cpb*_2_^+^		1	(1)	0	(0)	1.000

n: number of isolates; *E. coli*:* Escherichia coli*; *R. equi*: *Rhodococcus equi*; *C. perfringens*: *Clostridium perfringens*; *C. difficile*: *Clostridium difficile*; *∗*: absence of selected virulence markers studied; +: positive for the gene; -: negative for the gene; aEPEC: atypical enteropathogenic *E. coli*; STEC: Shiga toxin producing *E. coli*; aEAEC: atypical enteroaggregative *E. coli.*

*p* values less than 0.05 indicate significant differences between the urban and farm areas for the mono- or cooccurrence of bacteria(Fisher Exact test).

## Data Availability

The data used to support the findings of this study are included within the article.
